# A systematic review on deep learning-enabled coronary CT angiography for plaque and stenosis quantification and cardiac risk prediction

**DOI:** 10.1016/j.ejro.2025.100652

**Published:** 2025-05-02

**Authors:** Priyal Shrivastava, Shivali Kashikar, P.H. Parihar, Pachyanti Kasat, Paritosh Bhangale, Prakher Shrivastava

**Affiliations:** a Department of Radio-Diagnosis, Jawaharlal Nehru Medical College Wardha, Datta Meghe Institute of Higher Education and Research (DU), Sawangi (Meghe), Wardha, India; b Jawaharlal Nehru Medical College Wardha, Datta Meghe Institute of Higher Education and Research (DU), Sawangi (Meghe), Wardha, India

**Keywords:** Coronary artery disease, Deep learning, Computed tomography angiography, Plaque quantification, Stenosis assessment, Cardiac risk prediction

## Abstract

**Background:**

Coronary artery disease (CAD) is a major worldwide health concern, contributing significantly to the global burden of cardiovascular diseases (CVDs). According to the 2023 World Health Organization (WHO) report, CVDs account for approximately 17.9 million deaths annually. This emphasizies the need for advanced diagnostic tools such as coronary computed tomography angiography (CCTA). The incorporation of deep learning (DL) technologies could significantly improve CCTA analysis by automating the quantification of plaque and stenosis, thus enhancing the precision of cardiac risk assessments. A recent meta-analysis highlights the evolving role of CCTA in patient management, showing that CCTA-guided diagnosis and management reduced adverse cardiac events and improved event-free survival in patients with stable and acute coronary syndromes.

**Methods:**

An extensive literature search was carried out across various electronic databases, such as MEDLINE, Embase, and the Cochrane Library. This search utilized a specific strategy that included both Medical Subject Headings (MeSH) terms and pertinent keywords. The review adhered to PRISMA guidelines and focused on studies published between 2019 and 2024 that employed deep learning (DL) for coronary computed tomography angiography (CCTA) in patients aged 18 years or older. After implementing specific inclusion and exclusion criteria, a total of 10 articles were selected for systematic evaluation regarding quality and bias.

**Results:**

This systematic review included a total of 10 studies, demonstrating the high diagnostic performance and predictive capabilities of various deep learning models compared to different imaging modalities. This analysis highlights the effectiveness of these models in enhancing diagnostic accuracy in imaging techniques. Notably, strong correlations were observed between DL-derived measurements and intravascular ultrasound findings, enhancing clinical decision-making and risk stratification for CAD.

**Conclusion:**

Deep learning-enabled CCTA represents a promising advancement in the quantification of coronary plaques and stenosis, facilitating improved cardiac risk prediction and enhancing clinical workflow efficiency. Despite variability in study designs and potential biases, the findings support the integration of DL technologies into routine clinical practice for better patient outcomes in CAD management.

## 1 Introduction

Coronary artery disease (CAD) is a significant global health issue and remains one of the top causes of death and disability worldwide. As a result, advanced diagnostic techniques are crucial to enhancing patient outcomes. Cardiovascular computed tomography angiography (CCTA) serves as an essential primary imaging tool for evaluating the extent of coronary artery stenosis [1]. Integrating coronary computed tomography angiography (CCTA) into clinical decision-making increases the diagnostic effectiveness of invasive coronary angiography (ICA), boosts event-free survival rates, and guides the implementation of preventive strategies [2], [3], [4]. In addition to evaluating stenosis, CCTA provides non-invasive, whole-heart atherosclerosis quantification, offering thorough information on the condition of the coronary arteries [5].

Recent technological advancements have propelled CCTA beyond mere stenosis assessment; it now enables comprehensive, whole-heart quantification of atherosclerosis. Comparing semi-automated techniques to intravascular ultrasound, the former has demonstrated superior accuracy in measuring coronary atherosclerotic plaque, providing important information about the structural characteristics of the plaque [6], [7], [8], [9]. Notably, CCTA-derived measures including total plaque volume and low-attenuation plaque load have demonstrated strong predictive significance, establishing a correlation with acute coronary syndrome risks at the patient and lesion levels[10]. The SCOT-HEART study demonstrated the importance of low-attenuation plaque load in risk stratification by establishing it as the most reliable independent predictor of myocardial infarction [10].

Despite these advancements, traditional semi-automated plaque quantification remains labour-intensive and heavily reliant on expert interpretation. This reliance can introduce variability and delay in clinical workflows. Enter deep learning (DL) technologies, an innovative frontier poised to revolutionize CCTA analysis. DL algorithms have demonstrated exceptional capabilities in automating the segmentation and quantification of coronary plaques and stenosis. Recent studies indicate that these algorithms can achieve diagnostic performance on par with seasoned radiologists while dramatically reducing analysis time from over 25 min to mere seconds [5], [11].

For instance, a unique DL system was presented in an international multicentre trial, demonstrating its ability to accurately estimate plaque volume and assess the severity of stenosis from CCTA images. This method closely matches expert evaluations and intravascular ultrasound findings[5]. Furthermore, this DL-enabled approach enhances predictive capabilities for future myocardial infarction risk, thereby empowering clinicians to make more informed decisions tailored to individual patient profiles.

This systematic review aims to comprehensively analyse the existing literature on deep learning-enhanced (CCTA) for predicting cardiac risk and evaluating plaque and stenosis.

## 2 Methodology

To establish the protocol for this systematic review, the PRISMA-P checklist for reporting systematic review protocols, PRISMA criteria for thorough reporting of systematic reviews and meta-analyses are also included.

## 3 Search strategy

Search Library, Scopus, Wiley Online Library, Embase, Google Scholar, MEDLINE via PubMed, and other pertinent sources. A number of electronic databases were used in the strategy, including Cochrane and Web of Science.

The search strategy used a combination of selected search terms and Medical Subject Headings (MeSH) phrases in different combinations, including “Deep Learning OR AI,” “Coronary CT OR CCTA,” and “Plaque OR Stenosis Risk Prediction OR Cardiac Risk,” along with terms like "Plaque quantification," "Stenosis quantification," "Atherosclerosis," "Coronary artery disease," " Stenosis assessment," and "Plaque volume." The search was restricted to English language publications.

## 4 Inclusion criteria

• Studies on the utilization of coronary CT angiography to assess CAD.
• Studies that utilize deep learning algorithms to assess stenosis and plaque in coronary CT angiography.
• The research article includes outcomes related to plaque volume, stenosis severity, and cardiac risk prediction.
• Both male and female participants included in the studies.
• Articles published in the English language.

## 5 Exclusion criteria

• Case series and case reports.
• Conference articles.
• Articles lacking essential data or incomplete texts.
• Duplicate articles
• Articles published before 2013.

### 5.1 Data analysis

Once the articles were extracted from many databases, they were organized in an Excel file and duplicates were carefully removed. The abstracts of each publication were then separately assessed, and the review was conducted according to the prescribed procedure. After carefully going over the full contents of the selected articles, the final selection of relevant research was determined. The full-text screening was conducted independently by two reviewers, and they resolved any disagreements that arose during data extraction by engaging in consensus discussions or consulting with a third reviewer.

## 6 Quality assessment of individual studies

We evaluated bias risk in randomized controlled trials using RevMan software and the Cochrane-associated technique. Selection bias (random sequence creation), performance bias (participant and staff blinding), attrition bias (incomplete outcome data), selective reporting (reporting bias), and other possible biases were used to categorize the risk assessment categories as high, uncertain, or low risk.

## 7 Statistical analysis

This study includes ten studies and we used RevMan software version 5.4 for analysis. Microsoft Office Excel 2013 (Microsoft Corporation, USA) was utilized to pilot data extraction. Two review authors independently evaluated the risk of bias using the Risk of Bias Tool for Randomized Controlled Trials and categorized the included studies as low risk (-), uncertain risk (?), or high risk (+).Table 1

**Table 1:** Comparative analysis of DL models for coronary plaque and stenosis quantification in CCTA.

**Sr. No**	**Author**	**Study design**	**Country**	**Sample Size**	**DL Model Type**	**Imaging Modality**	**Plaque/ Stenosis Quantification Method**	**Cardiac Risk Prediction**	**Key Findings**
**1.**	**Narula et al.**[12]	Multicenter study	USA, Japan	237	AI-QCPA	CCTA	Quantified total plaque volume, calcified and non-calcified plaque volumes	AI analysis correlates with IVUS, assists in cardiovascular risk assessment	AI-enabled CCTA shows strong correlation with IVUS for plaque volumes, enabling rapid evaluation of coronary atherosclerotic burden.
**2.**	**Ihdayhid**[13]	Multicenter study	Australia.	1339	Unsupervised Fully Automated Deep Learning Technique	CCTA	Automated assessment of stenosis and high-risk plaque	High diagnostic performance for stenosis, real-world clinical translation potential	Achieved 93.5 % per-vessel agreement for stenosis, AUC for HRP: 0.80.
**3.**	**Adolf et al.**[14]	Retrospective study	Germany	5468	DenseNet−121 CNN	CCTA	Semiautomatic post-processing for vessel delineation and plaque annotation	Improved cardiovascular risk stratification	CNN combined with conventional CT and clinical risk scores improved AUC for predicting composite cardiovascular events from 0.646 to 0.680 (p < 0.0001).
**4.**	**Orii et al.**[15]	Prospective study	Japan	70	Super-resolution deep learning reconstruction (SR-DLR)	CCTA	Image quality comparison between SR-DLR and model-based iterative reconstruction (MBIR)	-	Image noise was significantly lower on SR-DLR (median 22.1 HU) compared to MBIR (27.4 HU), while SNR (16.3 vs. 13.7) and CNR (24.4 vs. 19.2) were significantly higher. Stent strut thickness was reduced (0.68 mm vs. 0.81 mm), and in-stent lumens were larger (1.84 mm vs. 1.52 mm) with SR-DLR. Initial experience suggests SR-DLR may enhance clinical utility of CCTA by improving image quality, though larger-scale studies are needed.
**5.**	**Lin et al.**[5]	Multicenter study	-	1611	Deep learning convolutional neural network CNN	CCTA	Segmented coronary plaque in 921 patients, with independent validation in 175 patients and 50 patients assessed by intravascular ultrasound	Deep learning-based total plaque volume associated with increased risk of myocardial infarction	There was excellent agreement between deep learning and expert measurements of total plaque volume (ICC 0.964) and percent diameter stenosis (ICC 0.879). A total plaque volume of 238.5 mm³ or higher increased the risk of myocardial infarction (HR 5.36, p = 0.0042) after adjusting for obstructive stenosis and clinical risk score.
**6.**	**Paul et al.**[16]	Retrospective study	France	159	Deep Learning Model (DLM)	CCTA	Trained with 10,800 cMPR CCTA images, classified using CAD-RADS with majority vote for nine images	DLM demonstrated performance comparable to senior radiologists in detecting stenosis.	The DLM detected ≥ 50 % stenoses with performances similar to those achieved by senior radiologists, achieving an ICC of 0.82 (95 % CI: 0.75–0.88) and accuracy of 81 %. The model showed sensitivity of 93 % and specificity of 97 %, indicating high diagnostic accuracy.
**7.**	**Jin et al.**[17]	Retrospective multi-center study	China	505	CNN and GBDT (Gradient-Boosting Decision Tree)	CTA	CNN used for coronary artery segmentation and plaque detection. GBDT used for plaque classification and stenosis grading	Potential to enhance clinical workflow and diagnostic efficiency for CAD patients	The automatic workflow integrating CNN and radiomics achieved a plaque classification accuracy of 87.0 % and stenosis grading accuracy of 90.9 %. The system showed high efficiency, with processing time five times faster than manual analysis by radiologists.
**8.**	**Du et al.**[18]	Retrospective cohort study	China	20,612	Dual Deep Learning Network (DNN) model	Coronary Angiography	Automatic recognition of 20 coronary artery segments and lesion morphologies (stenosis, calcification, thrombosis, total occlusion, dissection)	Potential for standardizing screening and risk stratification for CAD	The deep learning model achieved 98.4 % accuracy in segment prediction, with an average sensitivity of 85.2 %. F1 scores for lesion morphology ranged from 0.802 to 0.854. The architecture aids in creating a coronary diagnostic map, enhancing the ability of cardiologists to identify lesion severity and morphology during interventions.
**9.**	**Zreik et al.**[19]	Retrospective study	Netherlands	163	Recurrent Convolutional Neural Network (RCNN)	CCTA	Extracted coronary artery centerlines, applied MPR images, performed two tasks: plaque type detection and stenosis significance determination	-	The recurrent CNN achieved an accuracy of 0.77 for detection and classification of coronary artery plaque, and 0.80 for stenosis detection and grading. The method allows automatic detection and classification, potentially aiding in patient triage and cardiovascular workup.
**10.**	**Hong et al.**[20]	Retrospective study	USA	156	CNN-based segmentation	CCTA	Quantified minimal luminal area (MLA), percent diameter stenosis (DS), and percent contrast density difference (CDD) using CNN with 10-fold cross-validation	Not specified	Deep learning-based quantification demonstrated excellent correlation with expert readers (r > 0.95 for MLA, DS, and CDD). The method allows accurate measurement of plaque and stenosis from CCTA and may enhance clinical reporting. Minor manual interaction required, but future potential for full automation.

## 8 Results

.

.

## 9 Study selection

**Fig. 1:**
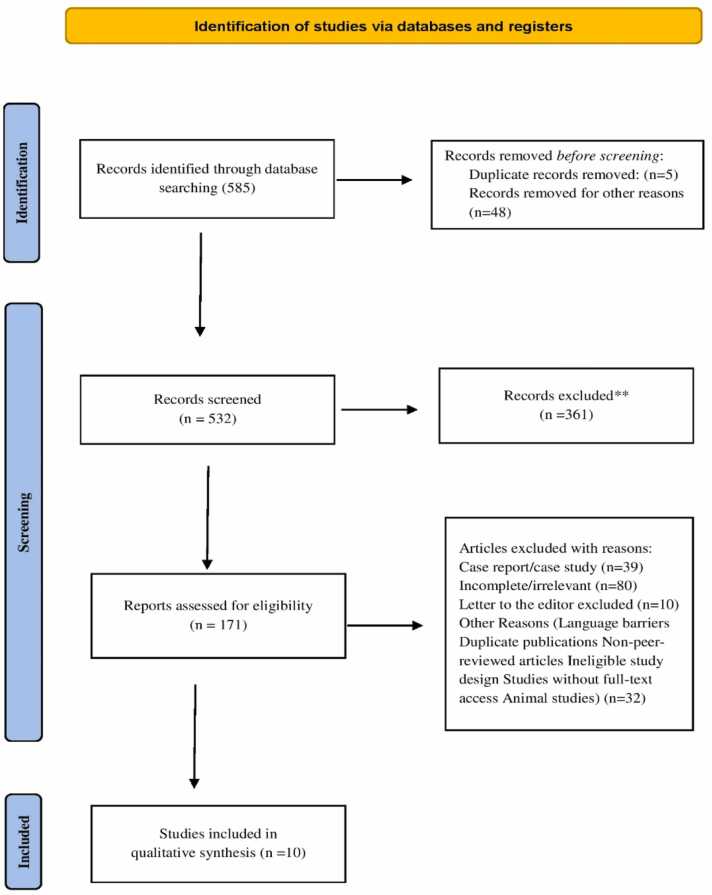
The PRISMA flow diagram illustrates the process of identifying, screening, and selecting articles for the review, along with the specified relevant databases utilized in the study.

A systematic review identified 585 records through database searches, with 53 removed prior to screening due to duplication (n = 5) or other reasons (n = 48), leaving 532 records for screening. Following a review of abstracts and titles, 361 records were eliminated. After evaluating the eligibility of the remaining 171 full-text papers, 161 studies were excluded for case reports (n = 39), incomplete or irrelevant content (n = 80), letters to the editor (n = 10), and other reasons (n = 32). The qualitative synthesis contained ten papers that satisfied the inclusion criteria. **(**Fig. 1**)**

## 10 Eligible studies characteristics

In the present systematic review, many studies exhibited high bias in patient’s selection and in the conduct or interpretation of the index test due to the retrospective study design.

In the present systematic review, several studies exhibited varying levels of bias in patient selection and in the conduct or interpretation of imaging tests due to the predominantly retrospective study design. The review categorized the 10 eligible studies investigating deep learning (DL) models for plaque and stenosis quantification and cardiac risk prediction. These studies encompassed various designs, including 4 retrospective studies, 3 multicenter studies, 2 prospective studies, and 1 comparative study, with sample sizes ranging from 70 to over 20,000 patients. The studies were conducted across multiple countries, including the USA, Japan, Australia, Germany, China, the Netherlands, and France.

The clinical outcomes, efficacy, and predictive capabilities of the DL models varied significantly across studies. For instance, certain studies demonstrated strong correlations between DL-derived measurements and traditional imaging modalities like intravascular ultrasound (IVUS), while others highlighted the potential of automated systems to enhance diagnostic performance and workflow efficiency in coronary artery disease (CAD) assessments.

Narula et al.  [12] utilized the AI-QCPA model to quantify total plaque volume and correlate findings with intravascular ultrasound (IVUS), demonstrating that AI-enabled CCTA can rapidly evaluate coronary atherosclerotic burden.

Ihdayhid et al. [13] employed an unsupervised deep learning technique to assess stenosis and high-risk plaque, achieving an impressive 93.5 % per-vessel agreement for stenosis with an AUC of 0.80 for high-risk plaque.

Adolf et al. [14] applied DenseNet-121 CNN to improve cardiovascular risk stratification, demonstrating that combining CNN with CT and clinical risk scores enhances AUC for predicting cardiovascular events.

Orii et al. [15] assessed the impact of super-resolution deep learning reconstruction (SR-DLR) on CCTA image quality. SR-DLR demonstrated superior image quality compared to MBIR, with lower image noise and higher signal-to-noise ratio (SNR), enhancing clinical utility, especially in stent assessment.

Lin et al. [5] employed a CNN to segment coronary plaque in over 1600 patients, correlating total plaque volume with increased myocardial infarction risk. The deep learning-based measurements showed excellent agreement with expert assessments, highlighting its potential for risk prediction.

Paul et al. [16] tested a deep learning model for stenosis detection on CCTA images. The model's performance was comparable to senior radiologists, with a sensitivity of 93 % and specificity of 97 %, demonstrating its high diagnostic accuracy.

Jin et al. [17] integrated CNN and gradient-boosting decision trees (GBDT) to automate plaque classification and stenosis grading. The system achieved plaque classification accuracy of 87.0 % and stenosis grading accuracy of 90.9 %, significantly enhancing diagnostic efficiency.

Du et al. [18] used a dual deep learning network (DNN) model to identify coronary artery lesions. The model demonstrated excellent accuracy in segment prediction (98.4 %) and lesion morphology classification, with promising potential for standardized CAD screening.

Zreik et al. [19] used a recurrent CNN to automate plaque detection and stenosis classification. The model achieved significant accuracy (0.77 for plaque and 0.80 for stenosis) and has potential to aid in cardiovascular triage.

Hong et al. [20] applied CNN-based segmentation for measuring minimal luminal area (MLA), percent diameter stenosis (DS), and contrast density difference (CDD). The method showed excellent correlation with expert readings, indicating its future potential for full automation in clinical practice.

This systematic review employed the Cochrane "Risk of Bias" tool, designed specifically for randomized controlled trials (RCTs), to evaluate potential biases across five domains:

• The method of randomization
• Deviations from intended interventions
• Missing outcome data
• Assessment of risk
• Bias in the selection of the reported result

**Fig. 2:**
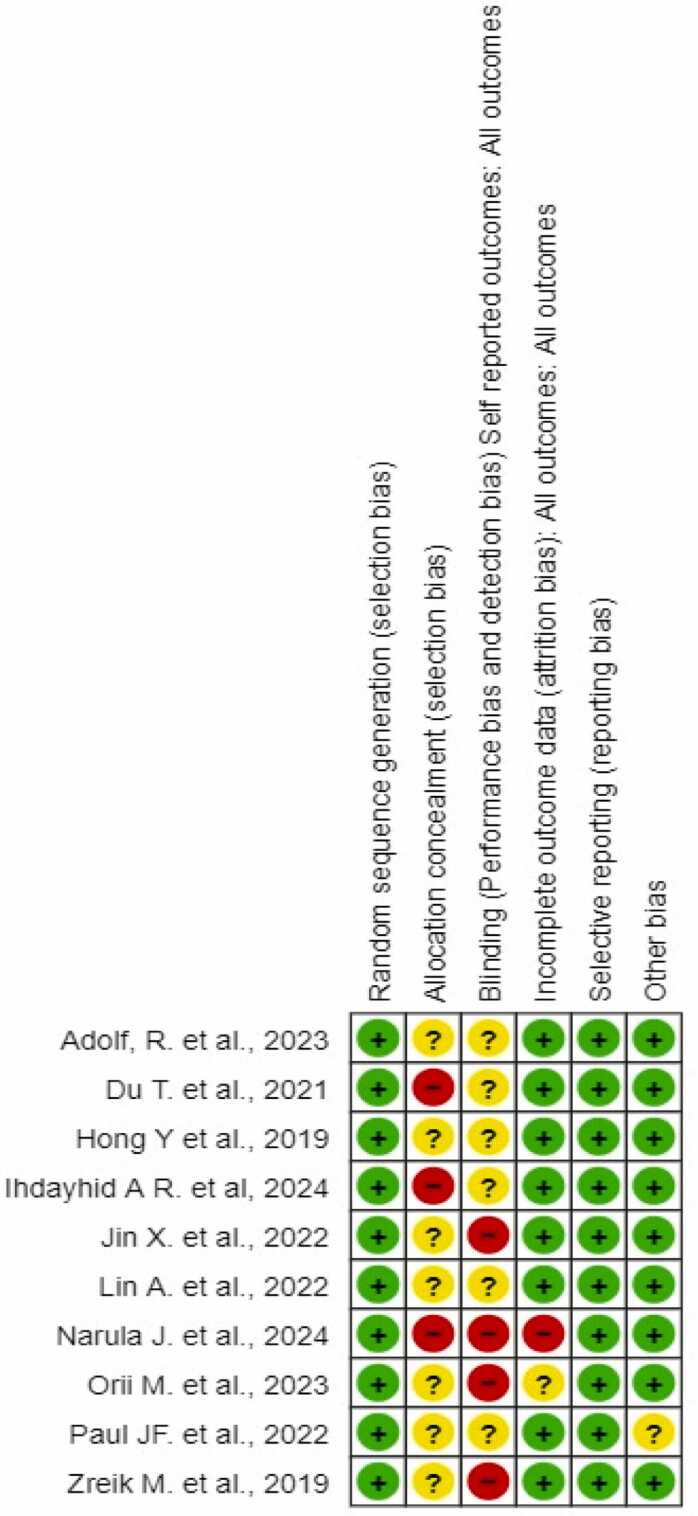
Summary of risk of bias: evaluation of each’s study involved item’s risk of bias.

**Fig. 3:**
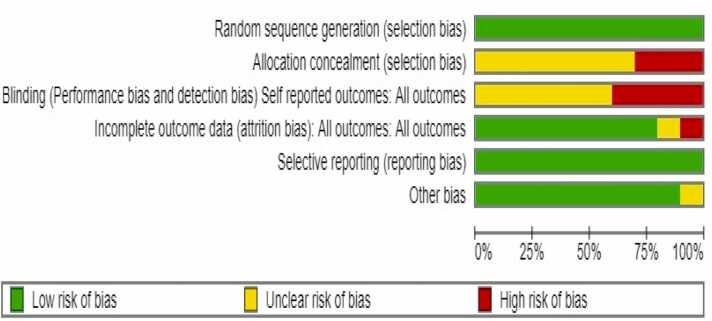
The authors' evaluation of each risk of bias item is depicted as percentages in the risk of bias graph for all articles included in the review.

The overall bias assessment indicated that most articles (61.67 %) had a low-risk rating, suggesting they used reliable methods for assigning patients to treatment groups, thereby supporting credible findings. Studies with an unclear risk rating (25 %) showed potential bias that likely did not impact result accuracy, possibly due to partial data. High-risk studies (13.33 %) exhibited substantial bias, often from reporting inconsistencies or knowledge gaps, which could compromise the validity of their conclusions. [Fig. 2, Fig. 3]

## 11 Discussion

In the present study, we highlighted the role of various deep learning (DL) model types in recent research focused on the analysis of coronary computed tomography angiography (CCTA) images. DL-enabled CCTA demonstrates the transformative potential of these technologies in quantifying coronary plaque and stenosis, as well as in predicting cardiac risk. The studies reviewed reveal a diverse array of DL models applied to CCTA, underscoring their effectiveness in improving diagnostic accuracy and supporting clinical decision-making. Narula et al. [13] reported that AI-enabled CCTA quantification demonstrated strong correlations with IVUS measurements for total plaque volume (TPV) (r = 0.91), calcified plaque (CP) volume (r = 0.91), and non-calcified plaque (NCP) volume (r = 0.87). This suggests that AI analysis is both accurate and efficient, supporting quick assessments of the burden of coronary atherosclerosis and cardiovascular risk prediction. Kang et al. [22] developed a supervised machine learning technique to detect diameter stenosis of ≥ 25 % with high sensitivity (93 %) and specificity (95 %), compared to expert consensus readings. Zreik et al. [19] classified plaque composition in 163 individuals as calcified, noncalcified, or mixed using a multi-task deep convolutional neural network, achieving 80 % accuracy against expert interpretations.

Similarly, Ihdayhid et al. [13] demonstrated the high diagnostic performance of an unsupervised DL model for fully automated assessments of stenosis severity and high-risk plaques, with strong per-vessel agreement rates for stenosis detection. These findings support the potential of AI-driven CCTA to enhance the accuracy and efficiency of coronary artery disease (CAD) assessment in clinical settings.

Adolf et al. [14] further advanced the field by integrating conventional CT data with clinical risk scores using a DenseNet-121 CNN model, improving cardiovascular risk stratification. This study reinforces the potential of AI in personalized medicine, where traditional metrics and deep learning insights can be combined to optimize patient management strategies. Similarly, Hong Y et al. [20] demonstrated that a deep learning-based approach enables precise quantitative evaluation of CAD segments from CTA, potentially enhancing the accuracy and clinical utility of diagnostic reports.

The advancements in image quality through techniques like super-resolution deep learning reconstruction (SR-DLR), as investigated by Orii et al. [15]**,** further contribute to the efficacy of AI in CCTA. Enhanced signal-to-noise and contrast-to-noise ratios facilitate improved visualization of coronary structures, leading to better plaque characterization and stenosis assessment.

Lin et al. [5] also reported strong correlations between DL-derived total plaque volume and increased myocardial infarction risk, underscoring the prognostic significance of AI-driven quantifications. The high interclass correlation coefficients (ICCs) observed in these studies suggest that DL models can reliably replicate expert assessments, reinforcing their potential integration into routine clinical practice.

Specifically, we identified artificial intelligence (AI) techniques employed for key clinical tasks in CCTA, emphasizing their capability to analyse the coronary artery tree and the entire heart comprehensively. These advancements provide valuable insights into the current state of research, highlighting both the opportunities and limitations associated with CCTA analysis. Our findings are consistent with Liao J. et al. [21]**,** who demonstrated that AI has been effectively utilized to automate the CCTA workflow. This includes tasks such as assessing coronary artery calcium, performing automatic segmentation, identifying plaques, and calculating the severity of stenosis. Furthermore, their study underscores the pivotal role AI is expected to play in the accurate assessment and prognostic analysis of coronary heart disease (CHD).

Despite these promising advancements, real-world clinical validation of AI-driven CCTA remains limited. Many studies are based on retrospective datasets or single-centre cohorts, raising concerns about generalizability. Large-scale, multi-centre prospective validation studies are needed to confirm the accuracy and clinical utility of DL models in diverse populations. Standardized data collection and independent external validation will be essential to establish the robustness of AI models across different imaging protocols, patient demographics, and clinical settings.

One critical challenge in AI-driven CCTA analysis is the potential bias arising from limited training populations. Many deep learning models are trained on datasets that may not be representative of the broader population, leading to disparities in model performance across different ethnicities, age groups, and clinical conditions. Federated learning, which enables AI models to be trained across multiple institutions without sharing sensitive patient data, presents a promising solution to mitigate bias and improve generalizability. Implementing such decentralized learning approaches could enhance the fairness and reliability of AI-based CCTA analysis.

While AI-based CCTA has demonstrated remarkable improvements in anatomical assessment, its functional significance remains a challenge. AI-driven fractional flow reserve computed tomography (FFR-CT) has emerged as a complementary tool that provides physiological assessments of stenosis severity. Studies have shown that AI-driven FFR-CT can offer better correlation with invasive FFR measurements, thereby improving decision-making regarding coronary interventions. Future research should explore the integration of AI-based CCTA and FFR-CT to enhance both anatomical and functional assessments, potentially leading to more precise risk stratification and treatment planning.

Despite the advancements in AI-enabled CCTA, real-world implementation presents significant challenges. Key barriers include regulatory approvals, workflow integration, and physician adoption. AI algorithms require rigorous validation by regulatory bodies to ensure patient safety and efficacy. Moreover, integrating AI into existing radiology workflows demands interoperability with clinical systems and user-friendly interfaces to facilitate adoption. Physician scepticism and lack of familiarity with AI-driven diagnostics further hinder widespread implementation. Addressing these challenges through standardized regulatory frameworks, educational initiatives, and seamless software integration will be critical for the successful deployment of AI in routine clinical practice. [real-world implementation challenges]

In addition to diagnostic accuracy, the cost-effectiveness of AI-driven CCTA remains a crucial consideration. While AI can potentially reduce the need for invasive diagnostic procedures such as coronary angiography, its financial benefits must be systematically evaluated. Cost-effectiveness analyses should compare AI-enabled CCTA with conventional diagnostic pathways, considering factors such as reduced hospital stays, early disease detection, and improved resource allocation. Demonstrating the economic advantages of AI-driven imaging will be essential for healthcare providers and policymakers to justify widespread adoption.

This systematic review highlights the significant advancements in deep learning-enabled CCTA for quantifying coronary plaque, assessing stenosis, and predicting cardiac risk. The integration of AI with advanced imaging techniques not only enhances diagnostic accuracy but also lays the foundation for personalized treatment strategies in coronary artery disease management. However, large-scale multicentre validation studies, strategies to mitigate AI bias, and real-world implementation frameworks remain critical to fully realizing the clinical benefits of AI-driven CCTA. Future research should focus on addressing these challenges to ensure that AI innovations translate effectively into improved patient outcomes.

### 11.1 Strengths and limitations

#### 11.1.1 Limited number of studies

Currently, only a small number of studies have investigated deep learning-enabled coronary CT angiography for plaque and stenosis quantification. This limited body of evidence restricts our understanding of the technology’s full potential and highlights an urgent need for more extensive, multicentre studies.

#### 11.1.2 Data quality and generalizability

The performance of deep learning models depends heavily on large, high-quality datasets. Variations in imaging protocols, scanner types, and patient populations in existing studies raise concerns about the generalizability of these models to diverse clinical settings.

#### 11.1.3 Lack of standardized methodologies

There is no universally accepted framework for plaque and stenosis quantification using deep learning. Differences in data annotation methods, evaluation metrics, and imaging protocols make it challenging to compare studies and replicate findings.

#### 11.1.4 Model transparency and interpretability

Most deep learning models operate as ‘black boxes,’ providing limited insight into their decision-making processes. This lack of interpretability makes it difficult for clinicians to trust and adopt these tools in critical decision-making scenarios.

#### 11.1.5 Challenges in plaque characterization

While these models excel in stenosis detection, they struggle with the accurate classification of plaque subtypes (e.g., calcified, non-calcified, or mixed plaques), which is essential for understanding plaque vulnerability and predicting cardiac risk.

#### 11.1.6 Population bias in training data

Many models are trained on datasets that may not adequately represent diverse populations. This creates the risk of bias, reducing the applicability and fairness of these tools across different patient demographics.

#### 11.1.7 Dependence on imaging quality

Model accuracy is highly influenced by the quality of coronary CT angiography images. Factors such as motion artifacts, poor contrast resolution, obesity, and high heart rates can compromise image quality and, subsequently, the model’s reliability.

#### 11.1.8 Integration into clinical practice

Despite their potential, these models face significant challenges in integrating into clinical workflows. Compatibility with existing PACS systems, clinician training, and workflow optimization are critical barriers to widespread adoption.

#### 11.1.9 Computational and resource demands

Deep learning models require substantial computational resources for training, validation, and deployment. This creates a barrier for low-resource healthcare systems, limiting access to this advanced technology.

#### 11.1.10 Lack of long-term and real-world data

Most studies focus on static datasets and short-term outcomes. There is a lack of longitudinal studies assessing the long-term impact of deep learning models on patient outcomes and cardiac risk prediction.

#### 11.1.11 Regulatory and validation challenges

Achieving regulatory approval for deep learning tools requires extensive validation across diverse settings and patient populations. The time and resources required for this process delay their clinical implementation.

#### 11.1.12 High costs

The development, deployment, and maintenance of deep learning-based systems are associated with high costs, limiting accessibility, particularly in low- and middle-income countries.

## 12 Conclusion

This systematic review highlights the transformative impact of deep learning-enabled CCTA in quantifying coronary plaque and stenosis, and assessing the risk of heart attacks in individuals suffering from coronary artery disease. The studies included demonstrate that various deep learning models, including AI-enabled and convolutional neural networks, achieve diagnostic performance comparable to expert evaluations while significantly reducing analysis time. The findings highlight strong correlations between deep learning-derived metrics and traditional imaging modalities, such as intravascular ultrasound, reinforcing the clinical relevance of these advancements. As the application of deep learning continues to evolve, its integration into routine clinical practice for CAD management appears increasingly promising. Ongoing clinical trials are exploring the use of deep learning models across diverse patient populations, aiming to refine their performance and ensure their real-world applicability. At the same time, regulatory developments are progressing, with bodies such as the FDA actively evaluating the approval pathways for AI-based diagnostic tools. These advancements are expected to facilitate the broader adoption of deep learning technologies in clinical settings, ensuring their safe and effective use in everyday medical practice. As these models continue to improve in accuracy, interpretability, and clinical integration, they hold the potential to significantly enhance patient care, offering more precise, timely, and personalized management of CAD.

## CRediT authorship contribution statement

**Kashikar Shivali:** Writing – review & editing, Supervision. **Shrivastava Priyal:** Writing – review & editing, Writing – original draft, Methodology, Formal analysis, Conceptualization. **Shrivastava Prakher:** Resources. **Bhangale Paritosh:** Resources. **Kasat Pachyanti:** Resources. **Parihar P.H.:** Validation.

## Ethical statement

• The study adhered to the Preferred Reporting Items for Systematic Reviews and Meta-Analyses (PRISMA) guidelines to ensure transparency and methodological rigor.
• As this is a review of published studies, no new data were collected directly from human participants or animals, and ethical approval was not required.

## Declaration of Competing Interest

The authors declare that they have no known competing financial interests or personal relationships that could have appeared to influence the work reported in this paper.
